# The clinical features, muscle pathology, and role of autophagy in anti-Ku-positive patients

**DOI:** 10.3389/fimmu.2025.1608735

**Published:** 2025-06-17

**Authors:** Lingya Qiao, Ying Lin, Mengyang Liu, Jiaqi Liu, Ke Li, Juan Chen, Qiang Shi

**Affiliations:** ^1^ Department of Neurology, Tianjin Huanhu Hospital, Tianjin, China; ^2^ Department of Neurology, The First Medical Center of Chinese PLA General Hospital, Beijing, China; ^3^ Department of Neurology, The Second Medical Center of Chinese PLA General Hospital, Beijing, China

**Keywords:** anti-Ku antibody, skeletal muscle pathology, autophagy, myositis, autoimmune disease

## Abstract

**Aims:**

This study aimed to examine the clinical and muscle histological characteristics of anti-Ku-positive patients. A preliminary investigation into the involvement of autophagy was conducted as well.

**Methods:**

Clinical characteristics, laboratory findings, and muscle histological features were collected from patients with isolated anti-Ku antibodies at the Department of Neurology, First Medical Center of the PLA General Hospital, between February 2011 to June 2024. Autophagy-related protein levels were semi-quantitatively assessed on muscle tissue samples using western blot (WB), with sporadic inclusion body myositis (sIBM) and immune-mediated necrotizing myopathy (IMNM) patients as comparison groups.

**Results:**

A total of 6 patients were recruited in the study (50% female, mean age at onset 47.6 ± 15.56 years, mean disease duration 7 ± 5.58 months). Extramuscular involvement was observed in most cases, including subcutaneous edema (33.3%), skin rash (33.3%), hyperpigmentation (33.3%), hair loss (33.3%), arthralgia (50%), and interstitial lung disease (ILD) (33.3%), etc. Coexisting connective tissue diseases included systemic sclerosis (SSc) (83.3%), systemic lupus erythematosus (SLE) (16.7%), and arthritis (16.7%). The distribution of muscle weakness was generally symmetrical and proximal (83.3%). Distal (50%) and axial (50%) muscle weakness could also be found. 2 patients exhibited peripheral nerve damage and myogenic damage in EMG, while 4 showed myogenic damage. Creatine kinase (CK) was mildly or moderately elevated. Muscle biopsy demonstrated two patterns: a neurogenic atrophy pattern and a myositis pattern characterized by a varying degree of necrotizing fibers (100%) with rimmed vacuoles (50%) or non-rimmed vacuoles (50%). Immunohistochemical (IHC) analysis revealed sarcolemma deposition of major histocompatibility complex class I (MHC-I) (83.3%) and MHC-II (83.3%), as well as predominant CD68-positive inflammatory infiltrates (66.7%). IHC for p62 revealed a sarcoplasmic punctate pattern (50%), along with a focal coarse staining pattern (50%) and occasional fine granular staining (33.3%). Electron microscopy (EM) demonstrated filamentous and lipid accumulation within vacuoles. WB analysis showed that p62 levels significantly differed between the anti-Ku and IMNM groups. Additionally, Parkin levels were highest in sIBM, while lysosome-associated membrane protein 2 (LAMP2) and microtubule-associated protein 1A/1B-light chain 3 (LC3) expression was highest in the anti-Ku-positive group in tendency.

**Conclusion:**

The muscular features were heterogeneous in anti-Ku-positive patients. A predominant myositis pattern was characterized by necrotizing fibers and vacuolar changes in muscle histology, which differ from sIBM and IMNM. Autophagy appeared to be a key mechanism implicated in the pathogenesis.

## 1 Introduction

Ku is a DNA-binding protein composed of two subunits weighing 70 and 80 kDa, which plays a significant role in the double-strand break DNA repair pathway in mammals (1). In 1981, Mimori et al. (2) first identified the anti-Ku antibody as a serological marker for the scleroderma-polymyositis (SSc-PM) overlap syndrome. Since then, it has been detected in various autoimmune diseases, including mixed connective tissue disease and certain malignancies, but rarely in healthy individuals (3). Currently, anti-Ku antibodies, classified as antinuclear antibodies (ANAs) and recognized as a type of myositis-associated autoantibodies (MAAs) (4) as well as a type of SSc-overlap autoantibodies (5).

Autophagy is an evolutionarily conserved, intracellular recycling pathway that preserves cellular homeostasis by clearing dysfunctional organelles, misfolded protein aggregates, and invading pathogens. Cytoplasmic cargo is first sequestered into double-membrane autophagosomes, which then fuse with lysosomes where the contents are degraded and the resulting macromolecules are recycled (6). Dysregulation of autophagy has been increasingly recognized in various autoinflammatory and autoimmune disorders, including Idiopathic Inflammatory Myopathies (IIMs) (7–9). Notably, impairments in lysosomal degradation pathways and mitophagy have been implicated in the pathogenesis of IIMs (9). Aberrant expression of core autophagy markers microtubule-associated protein 1A/1B-light chain 3 (LC3), p62/SQSTM1, lysosome-associated membrane protein 2 (LAMP2), and the mitophagy E3 ligase Parkin, together with ultrastructural findings of autophagic and rimmed vacuoles, indicates a stalled autophagic flux. Such impairment is thought to fuel persistent muscle inflammation, dysregulated immune activation, and progressive myofiber degeneration (7, 9–12).

The presence of anti-Ku autoantibody in IIMs is infrequent. It is predominantly observed in Scleromyositis (SM), a distinct clinical entity characterized by the coexistence of IIM and SSc (13–17). The spectrum of muscle involvement in anti-Ku autoantibody-positive myositis is heterogeneous (11, 18–27), especially the phenotype of myositis with isolated anti-Ku antibody remains poorly described. Recent studies have reported the presence of vacuolar changes in muscle biopsies from this subgroup, raising the possibility that autophagy dysregulation may contribute to a disease-specific pathomechanism (26). However, the precise role of autophagy in this subset remains largely undefined and warrants further investigation.

This study comprehensively analyzed the clinical manifestations and muscle histological features observed in patients with isolated anti-Ku antibodies who had undergone skeletal muscle biopsy at a Chinese Neuromuscular Disease Center. Additionally, we semi-quantitatively assessed autophagy protein and conducted a comprehensive literature review to identify key histopathological and clinical hallmarks associated with this condition.

## 2 Materials and methods

### 2.1 Ethics approval

The study received approval from the ethics committee of the First Medical Center of the PLA General Hospital (No. S2022-461-01). All patients enrolled signed an informed consent form.

### 2.2 Study population

We identified 16 patients who were labeled with anti-Ku antibodies positive in the muscle pathology database from the Neurology Department of the First Medical Center of the PLA General Hospital between February 2011 and June 2024. Following a reassessment of the MAAs and myositis-specific antibodies (MSAs), 10 cases were excluded due to the presence of additional MSA/MAA-positive or lower anti-Ku antibody titers. Ultimately, a total of 6 patients with isolated anti-Ku antibodies who had undergone muscle biopsy were included in the study. The antibody status of patients labeled as anti-Ku positive is provided in **Supplementary Table S1**. Autoantibodies directed against Ku antigen were evaluated by line blotting (EURO-LINE). Besides, 3 patients diagnosed with sporadic inclusion body myositis (sIBM) according to Lloyd-Greenberg criteria (28) and 6 patients diagnosed with immune-mediated necrotizing myopathy(IMNM) according to the criteria established by the European Neuromuscular Center (ENMC) (29) underwent western blot (WB) analysis of skeletal muscle tissue as control. IMNM patient was individually matched by age (within one year) and sex, while the sIBM patients were matched solely based on sex. Additionally, 1 healthy individual was included as a control.

### 2.3 Clinical data collection

Basic information, including age onset, gender, duration of disease, and intramuscular/extramuscular manifestations, was collected. Muscle strength was measured by the Medical Research Council (MRC) classification of Manual Muscle Testing (MMT). All patients were tested for MAAs and MSAs. Laboratory findings, including creatine kinase (CK) and lactate dehydrogenase (LDH)) as well as electromyography (EMG) and thigh magnetic resonance imaging (MRI) information, were also obtained.

### 2.4 Skeletal muscle pathology

All 6 anti-Ku antibody-positive patients underwent the muscle biopsy. We used muscle samples from both biceps and quadriceps with MRC grades 3 to 4. Muscle selection was also guided by clinical examination, EMG findings, and/or muscle MRI, to avoid end-stage fibrotic tissue and optimize histopathological yield. A summary of biopsy sites is provided in **Supplementary Table S1**. The muscle samples were rapidly frozen in liquid nitrogen after pre-cooling with isopentane and subsequently fixed. Frozen sections were prepared at a thickness of 8 μm. Routine histological and enzyme histochemical staining was performed, including hematoxylin-eosin (HE), modified Gomori trichrome staining (mGT), reduced coenzyme I (NADH-TR), succinate dehydrogenase (SDH), adenosine triphosphatase (ATPase), oil red O (ORO), etc. Additionally, serial 10 μm frozen muscle sections were processed for immunohistochemistry (IHC). Primary antibodies included membrane-attack complex (MAC), CD4, CD8, CD68, major histocompatibility complex class I (MHC-I), MHC class II, myxovirus resistance protein A (MxA), p62/SQSTM1, LC3, and LAMP2. The antibody specifications and full staining protocol are provided in **Supplementary Table S2**. For ultrastructural analysis, muscle biopsy samples were immediately fixed in 2.5% glutaraldehyde in 0.1 M phosphate buffer (pH 7.4) at 4°C for 4–6 hours. The specimens were then post-fixed in 1% osmium tetroxide (OsO_4_), dehydrated through a graded series of ethanol, and embedded in epoxy resin. Ultra-thin sections (70 nm) were cut using an ultramicrotome, mounted on copper grids, and stained with uranyl acetate and lead citrate. The sections were then examined under a transmission electron microscope (TEM) (HITACHI, HT7800) at an accelerating voltage of 80–120 kV.

### 2.5 Autophagy-related western blotting assay

WB analysis of skeletal muscle samples was performed using standard protocols in all 6 patients with isolated anti-Ku antibodies, 3 sIBM patients, and 6 IMNM patients. Protein extracts were prepared from muscle tissue and quantified using a bicinchoninic acid (BCA) assay. Equal amounts of total protein were separated by sodium dodecyl sulfate-polyacrylamide gel electrophoresis (SDS-PAGE) and transferred onto polyvinylidene fluoride (PVDF) membranes. The membranes were blocked with 5% non-fat milk or bovine serum albumin (BSA) and incubated overnight at 4°C with primary antibodies targeting p62, LC3, parkin, and LAMP. After washing, the membranes were incubated with horseradish peroxidase (HRP)-conjugated secondary antibodies. Protein signals were visualized using an enhanced chemiluminescence (ECL) detection system, and relative protein levels were normalized to GAPDH as a loading control.

### 2.6 Statistical analysis

Categorical variables were expressed as percentages and counts, while continuous variables were reported as means and standard deviations (SD). The variable CK, which exhibited a highly positive skew, was summarized using the median, first quartile (Q1), and third quartile (Q3) for descriptive purposes. Comparisons between two independent groups (anti-Ku myositis *vs*. sIBM, and anti-Ku myositis *vs*. IMNM) were performed using the Mann–Whitney U test due to the small sample size. All statistical analyses were conducted using GraphPad Prism (version 9, GraphPad Software), and p-values < 0.05 were considered statistically significant.

## 3 Results

### 3.1 Demographic and clinical information

In this study, 6 patients were identified, corresponding to an incidence rate of 0.5% for isolated anti-Ku antibodies among a cohort of 1,120 IIM patients from our muscle pathology database. Among these patients, 3 were female. The mean age of disease onset was 47.6 ± 15.56 years (range: 22–68 years). The duration of the disease ranged from 3 to 18 months, with a mean duration of 7 ± 5.58 months. **Tables 1**, **2** summarize the basic clinical characteristics and muscular involvement features of the six patients, respectively.

**Table 1:** Demographic and clinical characteristics of the anti-Ku myositis.

clinical features	Case 1	Case 2	Case 3	Case 4	Case 5	Case 6
Age (years old)	54	68	57	45	41	22
Gender (M/F)	M	M	F	M	F	F
Course (months)	6	18	6	3	6	3
ANA	1:100	1:1000	1:3200	1:1000	1:320	1:3200
Anti-dsDNA	–	–	–	–	–	–
Skin involvement						
	–	Skin rash predominantly in the abdomen and thighs, with hyperpigmentation and pruritus	Subcutaneous edema in four limbs, with hair loss	Hyperpigmentation in the abdominal region	Subcutaneous edema in four limbs	Red rash in the cheeks, chilblain-like skin lesions on ears, painful vesicular lesion at the bilateral distal hyperlaxity of fingers, with hair loss
Joint involvement						
-Arthritis	–	–	–	–	–	+
-Arthralgia	–	–	+	–	+	+
Lung involvement						
-ILD	–	+	–	–	+	–
System involvement						
-Fever	+	–	–	–	–	–
-Autoimmune diseases						
-SSc	–	+	+	+	+	+
-SLE	–	–	–	–	–	+
-Other	–	–	Nodular goiter with Hyperthyroidism Hepatitis B cirrhosis	Hepatitis B cirrhosis		Recurrent spontaneous abortion Parotid enlargement
-Tumor	Penile cancer	Papillary thyroid carcinoma	–	–	Papillary thyroid carcinoma with iodine‐131 therapy	

“+”, present; “–”, absent; “/”, not assessed. Gender: M, male; F, female.

ANA, antinuclear antibody; Anti-dsDNA, anti-double-stranded DNA antibody; ILD, interstitial lung disease; SSc, systemic sclerosis; SLE, systemic lupus erythematosus.

**Table 2:** Muscle involvement characteristics of the anti-Ku myositis.

clinical features	Case 1	Case 2	Case 3	Case 4	Case 5	Case 6
Peak CK(IU/L)	575	4000	1246	8563	1756	7629
Peak LDH(IU/L)	831	216	207	703	267	1869
Myalgia	–	–	+	+	–	+
Atrophy	+	–	–	–	–	–
Muscles involvement(MRC)						
-Neck flexor	5	5	5	3	4	3
-Neck extensor	5	5	5	4	3	3
-Deltoid	5/5	5-/5-	5-/5-	4/4	5-/5-	3/3
-Biceps	5/5	4/4	5-/5-	4/4	5-/5-	3/3
-Wrist extensors	5/5	4/4	5-/5-	5-/5-	5/5	3/3
-Quadriceps	5/5	5-/5-	4/4	4/4	4/4	3/3
-Gluteus maximus	5/5	5-/5-	4/4	4/4	4/4	3/3
-Ankle dorsflexors	4/4	4/4	5-/5-	5-/5-	5/5	3/3
-Pharyngeal	–	–	–	+	–	+
-Respiratory	–	–	–	–	+	+
EMG						
-Damage pattern	/ +	Myogenic damage	Myogenic damage	Myogenic damage	Myogenic damage	Myogenic damage
-Peripheral neuropathy		+	–	–	–	–
Thigh MRI						
-Fatty infiltration	/	+	/	+	+	/
-Fasciitis		–		–	–	
-Intramuscular oedema		+		+	+	

MRC, Medical Research Council scale (0–5); EMG, electromyography; MRI, magnetic resonance imaging. “+” = present; “–” = absent; “/” = not assessed. Muscle strength is scored using the MRC scale; values such as “5/5” indicate bilateral (right/left) strength.

CK, creatine kinase; LDH, lactate dehydrogenase; EMG, electromyogram.

#### 3.1.1 Extramuscular involvement

Extramuscular involvement was frequently observed among the study cohort. Of the 6 patients analyzed, 5 were diagnosed with SSc (83.3%) and 1 with systemic lupus erythematosus (SLE) (16.7%). Dermatological manifestations were predominant, affecting 5 patients (83.3%), and included subcutaneous edema, hyperpigmentation, and hair loss. Additionally, other clinical features were noted, with arthralgia (50%), interstitial lung disease (ILD) (33.3%), and arthritis (16.7%). Furthermore, several patients presented with comorbid autoimmune conditions, including nodular goiter with hyperthyroidism, recurrent spontaneous abortion, hepatitis B cirrhosis, and parotid gland enlargement. Notably, 3 patients had concurrent tumors, specifically penile cancer and papillary thyroid carcinoma.

#### 3.1.2 Muscular involvement

All patients exhibited muscle weakness, predominantly with a symmetrical and proximal distribution in both the upper and lower limbs (86.7%). Additionally, 3 patients (50%) presented significant distal weakness, with EMG confirming axonal neuropathy in 2 cases. Axial muscle weakness was observed in 3 patients (50%), including involvement of the pharyngeal and respiratory muscles. 1 patient exhibited diaphragm elevation (13.3%), while another one presented with scapular winging (13.3%). Furthermore, 3 patients reported myalgia (50%), and only 1 patient displayed muscle atrophy attributed to axonal neuropathy.

1 patient exhibited evidence of peripheral nerve damage, while another patient presented with peripheral nerve impairment accompanied by myogenic damage on EMG. Both patients have undergone lumbar MRI to exclude other potential causes of neurogenic impairment. The remaining 4 patients demonstrated myogenic damage alone. Among the 3 patients who underwent thigh MRI, muscle edema was observed in the affected areas, with no evidence of fasciitis. Serum CK levels were mildly to moderately elevated, with a median value of 2878 (1078,7863) U/L (range 575–8563 U/L, normal range 25–200 U/L). Similarly, the mean LDH level was elevated, with a mean value of 682.2 U/L (range 207–1869 U/L, normal range 109–245 U/L).

### 3.2 Skeletal muscle pathology

**Figure 1:**
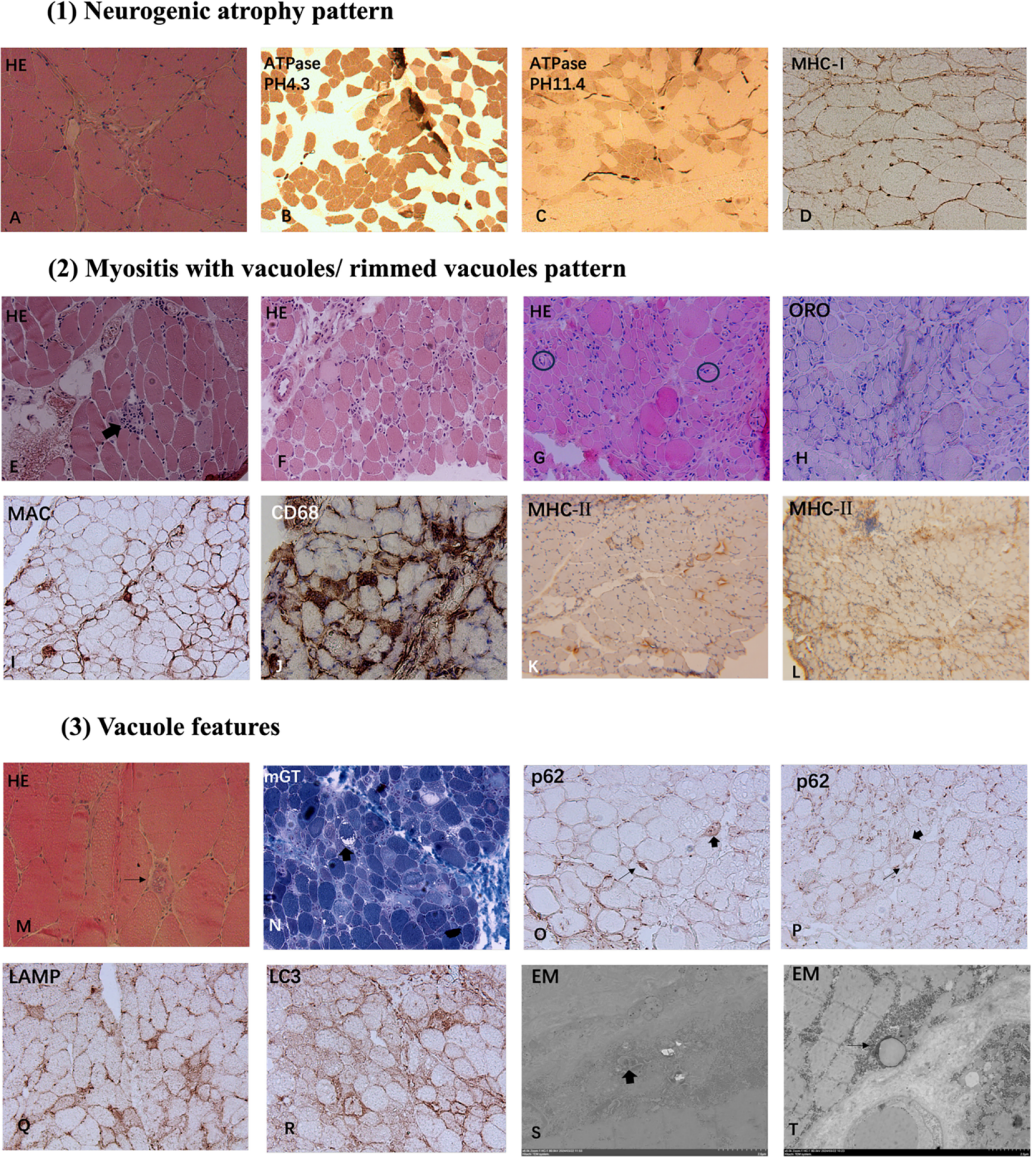
Myopathological Patterns in Anti-Ku Antibody-Positive Patients: (1) Neurogenic atrophy pattern: HE staining **(A)** reveals angular and irregular atrophic muscle fibers **(A)**. ATPase staining at pH 4.3 **(B)** and pH 11.4 **(C)** shows fiber-type grouping. Immunohistochemistry for MHC-I demonstrates sarcolemma deposition **(D)**. (2) Myositis with vacuoles/rimmed vacuoles pattern: HE staining shows necrosis and degeneration of myofibers, with some fibers showing phagocytosis (thick arrow, **E**), accompanied by vacuoles and rimmed vacuoles **(E–H)**. Markedly thickened endomysial capillaries is seen in some cases (black circles in **G**). Lipid deposition was visualized through ORO staining **(H)**. MAC sarcolemma deposition and CD68-positive macrophage endomysial infiltration are identified **(I, J)**. MHC-II expression is upregulated in sarcolemma and cytoplasm of some fibers **(K, L)** (3) Vacuole features: Several cases exhibit vacuolar pathology with rimmed vacuoles (thin arrow, M) and non-rimmed vacuoles (thick arrow, N). Most non-rimmed vacuoles display a more pronounced peripheral distribution. Immunohistochemical of p62 revealed a focal coarse pattern (thin arrows) and a sarcoplasmic punctate staining pattern (thick arrows) **(O, P)**. Immunostaining for LAMP2 and LC3 exhibited autophagic membrane-bound vacuoles **(Q, R)**. Analysis by EM demonstrated tubulofilamentous inclusion bodies and myelin figures in vacuolar localization (thick arrow, S) and lipid vacuoles enclosed by single-layer membranes (thin arrow, T). Magnification: x200 **(A, D, E, F, I, G, H–J, N–R)**; x100 **(B, C, K, L)**; x400 **(M)**; x6000 **(S)**; x5000 **(T)**. HE, hematoxylin-eosin staining; mGT, modified Gomori trichrome staining; ATPase, adenosine triphosphatase staining; ORO, oil red O staining; MHC-I, major histocompatibility complex class I; MHC-II, major histocompatibility complex class II; MAC, membrane-attack complex; LAMP2, lysosome-associated membrane protein 2; LC3, microtubule-associated protein 1A/1B-light chain 3; EM, Electron microscopy.

Skeletal muscle biopsies were performed on all 6 patients with detailed histological findings summarized in **Table 3**, **Figure 1**. A spectrum of pathological alterations was observed across all specimens. Notably, the predominant histological patterns included neurogenic atrophy and myositis characterized by the presence of vacuoles or rimmed vacuoles.

**Table 3:** Muscle histology features in anti-Ku antibody-positive patient.

pathological features	Case 1	Case 2	Case 3	Case 4	Case 5	Case 6
Muscle fibers						
-Neurogenic changes	+	+	–	–	–	–
-Regeneration	–	–	Many	Many	Some	Many
-Necrosis	Individual	Individual	Many	Many	Some	Many
-Phagocytosis	–	–	+	+	–	+
-Perifascicular atrophy	–	–	–	–	–	–
-Lipid deposition	–	–	+	++	–	–
-Thickened capillary	–	–	+	+	–	+
Vacuoles and autophagy markers						
-Vacuoles	–	–	Some	Some	–	Some
-RVs	–	Individual	–	–	Some	Some
-p62	–	Fine diffuse	Puncta staining	Fine diffuse and Puncta staining	Focal coarse	Puncta staining and Focal coarse
-LC3	–	–	Some	Some	–	Some
-LAMP	–	–	Some	Some	–	Some
Inflammatory infiltrates						
-CD4	–	–	–	+	–	+
-CD8	–	–	–	+	+	+
-CD68	–	+	+	+++	–	++
-MHC- I	–	+	+	++	+	+++
-MHC- II	–	+	+	++	+	++
-MAC sarcolemma deposition	–	–	–	+	–	++
- MAC capillary deposition	–	–	–	–	–	+
-MxA	–	–	–	–	–	–
EM						
-Lipid deposition	–	–	+	+	–	–
-Tubulofilamentous inclusion bodies and myelin figures	–	–	–	–	+	+

Semi-quantitative grading of abnormal fibers: none (0%), individual (>0% to <3%), some (3–10%), many (>10%).

Inflammatory infiltrates grading: - (0%), +(0%–10%), ++(10%–50%), +++(50%–100%) (27).

EM: “+” = present; “–” = absent.

RVs, rimmed vacuoles; MHC-I, major histocompatibility complex class I; MHC-II, major histocompatibility complex class II; MAC, membrane-attack complex; LAMP2, lysosome-associated membrane protein 2; LC3, microtubule-associated protein 1A/1B-light chain 3; MxA, myxovirus resistance protein A; EM, Electron microscopy.

#### 3.2.1 Neurogenic atrophy pattern

Muscle histological analysis of Cases 1 and 2 revealed characteristic features of neurogenic atrophy, including angular atrophic fibers, group atrophy, and fiber-type grouping. These findings indicate chronic neurogenic damage, suggestive of sustained denervation and subsequent compensatory reinnervation.

#### 3.2.2 Myositis featured necrotizing fibers and vacuoles

Excluding Case 1, the remaining patients exhibited a broad spectrum of myositis-like pathological features. Muscle biopsies revealed varying degrees of myofiber necrosis (100%) and degeneration (66.7%), with phagocytosis observed in select cases (50%). Notably, endomysial inflammatory infiltration was detected in 4 patients (66.7%), accompanied by sarcolemma and cytoplasm deposition of MHC-I (86.7%) and MHC-II (86.7%). Immunohistochemical analysis demonstrated that most infiltrating immune cells were CD68-positive (66.7%), with a subset expressing CD4 (33.3%) and CD8 (50%). Additionally, MAC was identified on the sarcolemma of non-necrotic muscle fibers (33.3%) and within capillaries (13.3%). In addition, 3 cases (50%) revealed thickened endomysial capillaries. Furthermore, ORO staining demonstrated lipid deposition in 2 cases (33.3%).

Muscle biopsies from several cases exhibited vacuolar pathology, with rimmed vacuoles identified in 3 cases (50%) and non-rimmed vacuoles in 3 cases (50%). Notably, most non-rimmed vacuoles displaying a more pronounced peripheral distribution. Immunohistochemical analysis of p62 revealed a focal coarse staining pattern associated with rimmed vacuoles, while other cases exhibited a small sarcoplasmic punctate staining pattern. Occasionally, some fibers displayed fine granular staining. Similarly, immunostaining for LC3 (50%) and LAMP2 (50%) demonstrated the presence of autophagic membrane-bound vacuoles, reinforcing the involvement of autophagic dysregulation in the pathological process.

#### 3.2.3 EM results

Analysis by EM demonstrated filamentous structures and myelin figures within vacuolar compartments. Additionally, lipid vacuoles enclosed by single-layer membranes were observed, suggesting potential metabolic alterations in the affected muscle fibers. These ultrastructural findings further support the involvement of autophagic dysregulation and degenerative mechanisms in muscle pathology.

### 3.3 Autophagy-related protein expression

**Figure 2:**
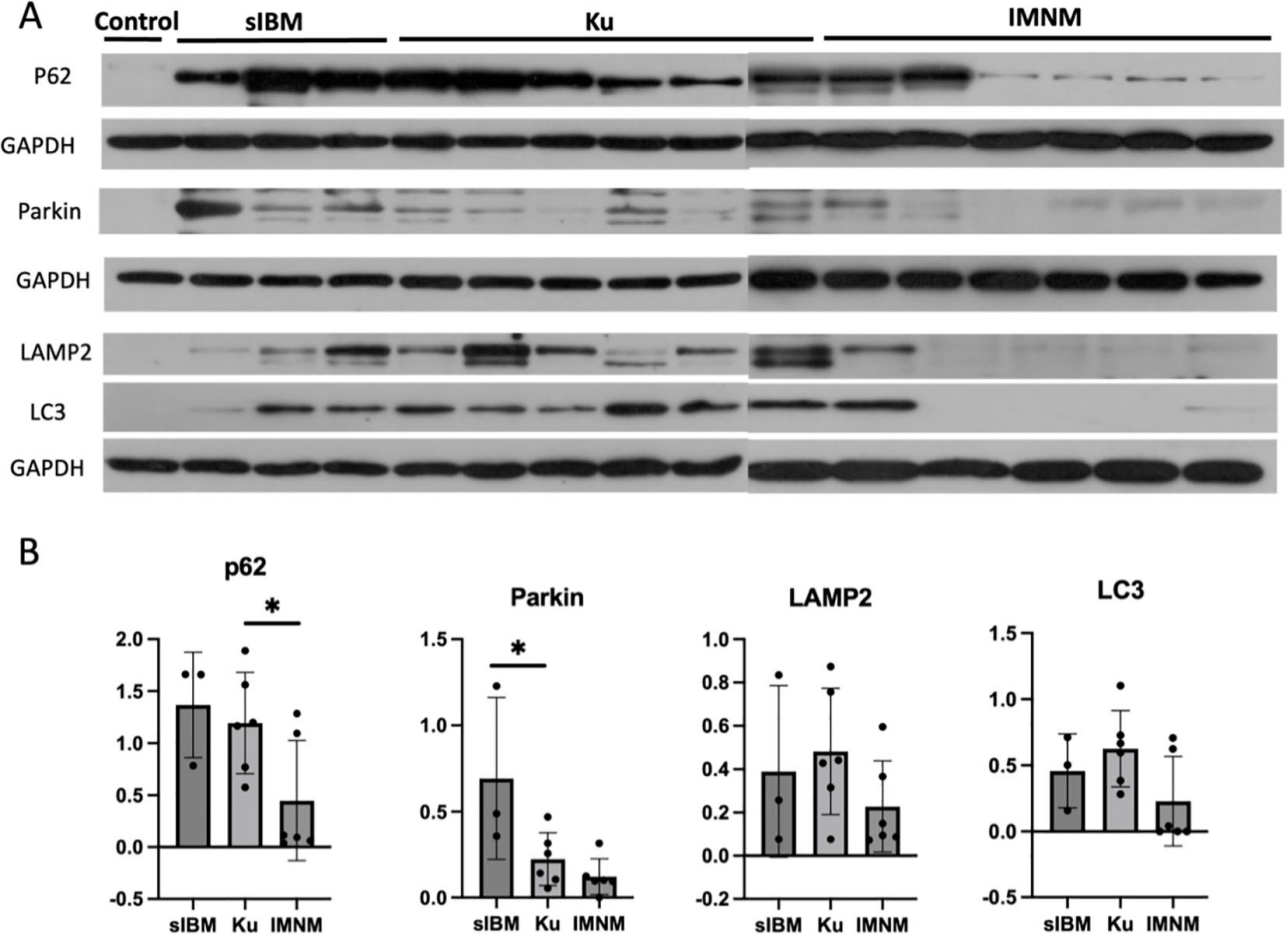
**(A)** Uncropped WB original images. **(B)** WB was semi-quantified using densitometry analysis by ImageJ, and quantified data were presented as mean ± SD. P62 levels significantly differed between the anti-Ku and IMNM groups. Parkin levels significantly differed between the anti-Ku and sIBM groups. LAMP2 and LC3 expression were highest in the anti-Ku-positive group, in tendency. (* p < 0.05). WB, western blots; LAMP2, Lysosome-associated membrane protein 2; LC3, microtubule-associated protein 1A/1B-light chain 3.

In light of the aforementioned pathological findings, autophagy dysfunction may play a role in the pathogenesis of this disease. To further investigate this hypothesis, WB was conducted for semi-quantitative evaluation of autophagy-related protein expression, including p62, LC3, parkin, and LAMP2, in muscle tissue. The expression profiles were also compared with those of patients diagnosed with sIBM and IMNM. A statistically significant increase in p62 protein levels was observed in the anti-Ku group compared to the IMNM group (p < 0.05). Notably, Parkin expression was significantly elevated in sIBM (p < 0.05). In addition, the expression levels of LAMP2 and LC3 tended to be highest in the anti-Ku-positive group, although the differences did not reach statistical significance. (**Figure 2**).

## 4 Discussion

We analyzed 6 patients with isolated anti-Ku antibodies—just 0.5% of the 1,120 IIM cases seen at our center—highlighting the exceptional rarity of anti-Ku myositis. Among them, 5 patients have been diagnosed with SSc, consistent with previous reports (3, 18). Additionally, our findings highlight the frequent occurrence of extramuscular manifestations in patients with isolated anti-Ku autoantibodies. Previous studies have reported a higher prevalence of SLE comorbidity among patients in the USA, whereas research from Japan reports a greater incidence of SSc and myositis (30, 31). Our findings suggest that the disease distribution in China more closely resembles that observed in Japan. These results support the hypothesis that the presence of anti-Ku antibodies may be associated with distinct disease manifestations across different ethnic groups.

The pattern of muscle weakness in our cohort was symmetrical and proximal, affecting both the upper and lower limbs consistent with other IIMs. Additionally, axial muscle weakness was observed in a subset of patients. Head drop syndrome and/or camptocormia, which indicated axial involvement, had been reported in SSc-PM patients as a main character (32) as well as in anti-Ku-positive patients (20, 21, 23). Furthermore, we identified 3 patients who exhibited an atypical weakness pattern characterized by distal muscle involvement, which is uncommon in myositis. Further evaluation revealed axonal damage in 2 of these patients, while 1 exhibited distal weakness without motor nerve involvement. Previous studies suggest that distal weakness may be a distinguishing feature of anti-Ku myositis compared to other forms of myositis (19). Further studies are required to better characterize the pattern of muscle weakness in anti-Ku-positive patients.

To further investigate the muscle pathological features of isolated anti-Ku positive patients, we reviewed the existing literature on muscle histology and summarized the findings in **Table 4**. Previous studies have demonstrated that this autoantibody is primarily associated with a necrotizing pattern in muscle biopsy findings (18, 19, 22, 24, 27). Additionally, rimmed vacuoles or vacuoles were documented in a subset of this particular cohort (21, 23, 26). Our study identified two distinct pathological patterns: neurogenic atrophy and myositis with vacuoles/rimmed vacuoles pattern. A review of prior studies suggests that myositis associated with anti-Ku antibodies exhibits a higher degree of neurogenic damage compared to other antibodies linked to SSc (18). Moreover, multiple reports have documented peripheral nerve palsy in individuals with anti-Ku antibodies (20, 33, 34). In our study, 2 patients displayed peripheral polyneuropathy as evidenced by EMG in addition to the neurogenic atrophy pattern found in muscle pathology. These findings suggest that peripheral nerve involvement may be a key clinical feature associated with anti-Ku autoantibodies. The observed myositis pattern featured varying degrees of myofiber necrosis and degeneration, accompanied with endomysial macrophages-predominant lymphomonocytic inflammation and MAC sarcolemma deposition, resembling IMNM. However, notable distinctions were observed, including MHC-II deposition and the presence of vacuoles, including rimmed vacuoles, were identified, which are atypical for IMNM. In recent years, the concept of SM has been proposed to describe myositis associated with SSc (35). The clinical-sero-pathological approach has been recognized as a well-established and effective method for evaluating myositis. Among the antibodies associated with SM, anti-Ku is one of the key autoantibodies. Additionally, capillaropathy, a pathological feature previously suggested to be characteristic of SM (36, 37), was found in 3 patients. Nevertheless, endomysial fibrosis, another recognized characteristic of SM (32), was not definitively observed in our cohort. These findings indicate that SM may exhibit distinct clinicopathological features depending on the specific antibodies present. Further studies are needed to better characterize the muscle pathology associated with anti-Ku-positive SM.

**Table 4:** Muscle pathology features of anti-Ku antibody-myositis from previous literature study.

No.	Year	Country	Number of patients	Muscle pathology features
1	2012	France	11	1 case of neurogenic muscular atrophy, 2 cases of rimmed vacuolar myopathy, and the rest of IIM
2	2016	Japan	1	PM
3	2017	France	1	IIM
4	2018	Japan	1	IIM with rimmed vacuole
5	2020	China	7	6 cases of NM and 1 case of IIM
6	2020	France	8	7 cases of NM, 1 case only showed MHC- I upregulation
7	2021	USA	4	3 cases of NM and 1 case of scattered muscle fiber degeneration
8	2024	Japan and Germany	26	Necrotizing myopathy features with MHC class II expression and clusters of perivascular inflammatory cells.
9	2024	Germany	26	Diffuse sarcolemmal MHC-I and -II immunoreactivity, myofiber necrosis, endomysial inflammation, thickened capillaries, and vacuoles. Protein aggregates in EM.

IIM, idiopathic inflammatory myopathy; NM, necrotizing myopathy; MHC-I, major histocompatibility complex class I; MHC-II, major histocompatibility complex class II; EM, Electron microscopy.

We highlighted the presence of vacuoles and autophagy-associated protein deposition in muscle histology. The observed vacuoles were primarily located at the periphery of muscle fibers. IHC for p62 revealed a focal coarse staining pattern, along with a sarcoplasmic punctate pattern and occasional fine granular staining; IHC for LAMP2 and LC3 demonstrated autophagic membrane-bound vacuoles. Additionally, through WB analysis, our findings indicate that p62 levels were significantly elevated in the anti-Ku-positive group but remained lower than those observed in sIBM. Furthermore, Parkin expression was highest in sIBM, while LAMP2 expression tended to be highest in the anti-Ku-positive group. Previous studies have established that p62 staining is a valuable diagnostic marker for sIBM (38), its focal enrichment—together with autophagy markers restricted to vacuoles—points to defective autophagosome maturation at a late stage (10, 12). Moreover, abnormal mitophagy has been identified in sIBM, as evidenced by the presence of COX-negative fibers, indicating mitochondrial dysfunction. Parkin, a key protein associated with mitophagy, was most highly expressed in the sIBM group, consistent with previous findings (7). By contrast, LAMP2, a glycoprotein essential for lysosomal adhesion, has been reported as low expression in sIBM in prior studies (12). Interestingly, LAMP2 and LC3 expression in the anti-Ku-positive group tended to be the highest, suggesting that lysosomal degradation disorders may contribute to anti-Ku-associated myositis, distinguishing it from sIBM. The detection of lipid deposition via ORO staining further supports the presence of lysosomal degradation abnormalities. Recent proteomic and transcriptomic profiling of anti-Ku myositis identified marked activation of autophagy, proteasome, and hnRNP-linked stress pathways, together with sarcoplasmic aggregates containing p62, BAG3, myotilin and immunoproteasome β5i (26). Since LAMP2 and LC3 are involved in both autophagy and proteasomal degradation, our findings further support this concept. Mechanistically, heterogeneous nuclear ribonucleoproteins (hnRNPs) regulate mRNA processing and interact with the Ku70/Ku80 heterodimer within the non-homologous end-joining (NHEJ) complex (39, 40). Cellular stress or Ku70 deficiency disrupts this interaction, compromises DNA repair and telomere integrity (41). As an integral component of the DNA-dependent protein-kinase (DNA-PK) holoenzyme, Ku70 assembles stress-responsive ribonucleoprotein condensates with HEXIM1 that dampen type I interferon (IFN-I) signaling (42) and modulate phosphorylation of substrates such as hnRNP-U during the DNA double-strand break response (43). IFN-I is a potent inducer of CIITA and therefore of MHC-II expression, offering a plausible explanation for the aberrant HLA-DR up-regulation on myofibers in our cohort (44). Ku70 also limits apoptosis by sequestering the pro-apoptotic factor Bax; its loss permits Bax ubiquitination, mitochondrial depolarization, Parkin recruitment, and heightened mitophagic demand (45, 46). Activated Bax in turn triggers LC3-dependent autophagy (47), linking Ku deficiency to heightened autophagic demand. When lysosomal capacity is exceeded, Parkin-labelled organelles and p62-bound aggregates accumulate within LC3-positive vacuoles that fail to mature, aggravating myofiber damage. Recent data further demonstrate that myositis-associated autoantibodies can be internalized by muscle fibers and impair the function of their cognate antigens (48). Collectively, these findings support a model in which Ku70 dysregulation—and possibly anti-Ku autoantibodies—intersects with the hnRNP network, lysosomal systems, and LC3-mediated autophagy. The resultant defects in DSB repair, protein quality control, and aggregate clearance may converge to drive the pathology observed in anti-Ku–positive patients. Although our findings suggest that anti-Ku-positive myositis differs from sIBM, we identified 1 patient exhibiting typical muscle pathology consistent with sIBM. A fine granular pattern of autophagy markers throughout the entire sarcoplasm indicates the early autophagy stage impairment (7). Our study found that some fibers also displayed fine granular staining. These observations raise the possibility that different stages of autophagy may contribute to disease heterogeneity. Future work should dissect stage-specific autophagy defects and clarify how anti-Ku antibodies intersect with Ku70, hnRNP networks, and lysosomal function to drive muscle degeneration.

This study has several limitations. Firstly, the small sample size is partly due to the low incidence of the condition. Consequently, our findings require validation in larger, multicenter studies. Secondly, WB analyses included only one healthy-muscle sample, used solely as a qualitative reference for band intensity and excluded from statistical testing. Although this limits quantitative interpretation, it does not influence the reported statistics. Thirdly, preliminary experiments suggest a potential involvement of autophagy in the pathogenesis. WB is intrinsically semi-quantitative; accordingly, the protein data presented here represent relative, not absolute, abundance. A further limitation is methodological heterogeneity in autoantibody detection. Although line blot balances feasibility with specificity, we acknowledge that different platforms vary in sensitivity. Future multicenter efforts should apply a single high-specificity assay to larger cohorts.

## 5 Conclusion

The muscle involvement was heterogeneous in isolated anti-Ku antibody-positive patients. Our study identified two distinct pathological patterns in skeletal muscle: a neurogenic atrophy pattern and a myositis pattern characterized by varying degrees of necrotizing fibers with rimmed or non-rimmed vacuoles. Autophagy has emerged as a key mechanism implicated in the pathogenesis of this condition. This is the first study on autophagy in anti-Ku antibody patients in Asia. Notably, anti-Ku-associated myositis differs from both sIBM and IMNM in multiple aspects. This work is a preliminary, descriptive case series designed to generate hypotheses that can be tested in larger, multi-center cohorts. Further investigations in larger cohorts are warranted to better characterize isolated anti-Ku antibody-positive patients’ clinical and pathological features and elucidate their underlying mechanisms.

## Data Availability

The raw data supporting the conclusions of this article will be made available by the authors, without undue reservation.
