# Supplementary material for: The clinical features, muscle pathology, and role of autophagy in anti-Ku-positive patients
Source: Front Immunol. 2025 Jun 17;16:1608735. doi: 10.3389/fimmu.2025.1608735 (PMC12209196; doi:10.3389/fimmu.2025.1608735)
Supplement: Supplementary file 1 [file DataSheet1.docx]

Table S1 Antibody status and biopsy site of patients labeled as anti-Ku positive

| Patient ID | Age | Sex | Anti-Ku antibody | MSAs | Other MAAs | Biopsy site |
| --- | --- | --- | --- | --- | --- | --- |
| P1 | 50 | F | ++ | Jo-1(++) | Ro52(+) | L-D |
| P2 | 40 | F | ± |  |  | R-BB |
| P3 | 63 | F | ++ | HMGCR(++) |  | L-QF |
| P4 | 45 | M | ++ |  |  | L-BB |
| P5 | 65 | F | ± |  | Ro52(++) | R-BB |
| P6 | 17 | M | + | Mi-2(+++) |  | R-D |
| P7 | 41 | F | +++ |  |  | R-QF |
| P8 | 18 | M | ++ | HMGCR(+++) |  | L-BB |
| P9 | 73 | F | + | SAE(++) |  | R-D |
| P10 | 54 | M | ++ |  |  | L-QF |
| P11 | 57 | F | +++ |  |  | L-QF |
| P12 | 41 | F | ± |  |  | L-BB |
| P13 | 29 | F | +++ | MDA5(+++) | Ro52(++) | R-BB |
| P14 | 68 | M | ++ |  |  | L-BB |
| P15 | 40 | F | ± | cN-1A(±) |  | R-QF |
| P16 | 22 | F | ++ |  |  | R-QF |

This table summarizes the autoantibody profiles of patients identified as anti-Ku positive in the muscle sample database. Patients highlighted in yellow represent the final cohort included in the analysis. Anti-Ku positivity was confirmed by the EUROLINE line blot. Other myositis-specific and associated antibodies are shown as well.

The intensity of antibody staining was semi-quantitatively graded as follows: ± = equivocal, + = weakly positive, ++ = positive, +++ = strongly positive.

Biopsy site abbreviation: QF, quadriceps femoris; BB, biceps brachii; D, deltoid; R-, right; L-, left.

| antibody | clone | company |
| --- | --- | --- |
| MHC-Ⅰ (HLA-A, B, C) Ab-3 | W6/32 | Thermo Fisher Scientific |
| MHC-Ⅱ | B308 | Affinity Bioreagents |
| Anti-Human C5b-9 (MAC) | Ab55811 | Abcam |
| p62 (SQSTM1) | Ab207305 | Abcam |
| LAMP | Ab199946 | Abcam |
| LC3 | Ab48394 | Abcam |
| MxA | ZRB2592 | Merck |
| Anti-Human CD4 | Ab133616 | Abcam |
| Anti-Human CD8 | Ab217344 | Abcam |
| Anti-Human CD68 | Ab213363 | Abcam |

Table S2 Antibodies used in this work

Immunohistochemical staining was performed on 8–10 μm cryosections mounted on poly-L-lysine–coated glass slides. Sections were air-dried, fixed in cold acetone for 10 minutes at –20°C, and rehydrated in phosphate-buffered saline (PBS, pH 7.4). Endogenous peroxidase activity was quenched using 3% hydrogen peroxide for 10 minutes at room temperature. Non-specific binding was blocked with 10% normal goat serum for 30 minutes. Slides were incubated overnight at 4°C with primary antibodies diluted in PBS containing 1% BSA. The primary antibodies used is showed in the table. After washing, sections were incubated with appropriate biotinylated secondary antibodies for 30 minutes. Diaminobenzidine was used as chromogen, and sections were counterstained with hematoxylin.
